# Expression gradient of metalloproteinases and their inhibitors from proximal to distal segments of abdominal aortic aneurysm

**DOI:** 10.1007/s13353-021-00642-3

**Published:** 2021-06-06

**Authors:** Aleksandra Augusciak-Duma, Karolina L. Stepien, Marta Lesiak, Ewa Gutmajster, Agnieszka Fus-Kujawa, Malwina Botor, Aleksander L. Sieron

**Affiliations:** 1 Department of Molecular Biology, Faculty of Medical Science in Katowice, Medical University of Silesia, Katowice, Poland; 2 Department of Medical Genetics, Faculty of Medical Science in Katowice, Medical University of Silesia, Katowice, Poland

**Keywords:** Metalloproteases, Abdominal aortic aneurysm, Angiogenesis, Remodelling, Extracellular matrix

## Abstract

Abdominal aortic aneurysm refers to abnormal, asymmetric distension of the infrarenal aortic wall due to pathological remodelling of the extracellular matrix. The distribution of enzymes remodelling the extracellular matrix and their expression patterns in the affected tissue are largely unknown. The goal of this work was to investigate the expression profiles of 20 selected genes coding for metalloproteinases and their inhibitors in the proximal to the distal direction of the abdominal aortic aneurysm. RNA samples were purified from four lengthwise fragments of aneurysm and border tissue obtained from 29 patients. The quantities of selected mRNAs were determined by real-time PCR to reveal the expression patterns. The genes of interest encode collagenases (*MMP1*, *MMP8*, *MMP13*), gelatinases (*MMP2*, *MMP9*), stromelysins (*MMP3*, *MMP7*, *MMP10*, *MMP11*, *MMP12*), membrane-type MMPs (*MMP14*, *MMP15*, *MMP16*), tissue inhibitors of metalloproteinases (*TIMP1*, *TIMP2*, *TIMP3*, *TIMP4*), and ADAMTS proteinases (*ADAMTS1*, *ADAMTS8*, and *ADAMTS13*). It was found that MMP, TIMP, and ADAMTS are expressed in all parts of the aneurysm with different patterns. A developed aneurysm has such a disturbed expression of the main participants in extracellular matrix remodelling that it is difficult to infer the causes of the disorder development. *MMP12* secreted by macrophages at the onset of inflammation may initiate extracellular matrix remodelling, which, if not controlled, initiates a feedback loop leading to aneurysm formation.

**Supplementary Information:**

The online version contains supplementary material available at 10.1007/s13353-021-00642-3.

## Introduction

Abdominal aortic aneurysm (AAA) is an abnormal, asymmetric distension of the infrarenal aortic wall of 3 cm or greater (Keisler and Carter 2015). The enlargement affects the three layers of the aorta. The condition is asymptomatic, and when the patient is not undergoing ultrasound for other indications, the first symptom is aortic dissection, which may lead to the patient’s death (Sakalihasan et al. 2005). An increased risk of developing AAA is strongly correlated with gender, age, smoking, family history of AAA, atherosclerotic disease, spinal cord injury, and genetic predisposition (Lederle et al. 2000; Li et al. 2013; Sakalihasan et al. 2005). AAAs are the major cause of morbidity and mortality in ageing societies (Humphrey and Holzapfel 2012). Specifically, in the overall European population, the prevalence is 4.3–7,1%, with 80% mortality resulting from AAA rupture (Li et al. 2013) (Hohneck et al. 2019). In the Polish population aged over 65 years, the incidence of AAA is 2.62% and almost 4 times higher in men (4.32%) than in women (1.23%) (Mikołajczyk-Stecyna et al. 2013; Tkaczyk et al. 2019).

Aneurysms develop as a result of degeneration of the arterial media and elastic tissues (Keisler and Carter 2015). The pathogenesis of AAA involves numerous processes, including inflammation, apoptosis of vascular smooth muscle cells (VSMCs), degradation of extracellular matrix (ECM), and oxidative stress (Mikołajczyk-Stecyna et al. 2013; Tkaczyk et al. 2019).

Proteases that facilitate ECM remodelling, cell migration and invasion, and the turnover of growth factors are one of the most important factors for the development of AAAs (van Hinsbergh et al. 2006). Matrix metalloproteinases, particularly *MMP2* and *MMP9*, were found to be elevated in the affected tissue. An imbalance between proteases and their inhibitors results in disruption of the homeostasis between the synthesis and degradation of the ECM (van Hinsbergh et al. 2006; Kadoglou and Liapis 2004; Nosoudi et al. 2015; Plaisier et al. 2004; Wilson et al. 2006).

The expression of genes responsible for ECM remodelling changes due to inflammation, which is a stimulus for the initiation of the process of abdominal aortic aneurysm formation. The analysis of individual parts of the aneurysm, rather than the analysis of the entire modified tissue in comparison with the control tissue, may reveal genes whose expression disturbances are responsible for the pathological growth of aortic tissue. This work was focused on the analysis of the expression profiles of 20 selected genes in arbitrary defined segments along surgically removed AAAs.

## Materials and methods

### Patient characteristics

A total of 29 samples from 3 females and 26 males were collected following AAA surgery from patients who were scheduled for open aortic repair (OAR). The patients who underwent surgery for AAA included in this study were of both sexes, ranging in age from 57 to 82 years (mean 67.5 ± 6.35 years). The AAA patients excluded from the study were those who fulfilled the following criteria: (a) chronic obstructive pulmonary disease (COPD); (b) diabetes; (c) creatinine level > 1.0; (d) reconstruction of coronary vessels and thoracic aorta (CABG); (e) reconstruction of carotid artery (ICA); (f) diagnosed generalized atherosclerosis (AO); (g) family history of AAA or inherited cardiovascular syndromes; and (h) lack of ability to provide informed consent for surgical treatment. The research plan was approved by the Bioethics Committee of the Medical University of Silesia in Katowice, protocol no. KNW/0022/KB1/55/14 and its further extension no. KNW/0022/KB1/55/1/14/17.

### Materials

**Fig. 1:**
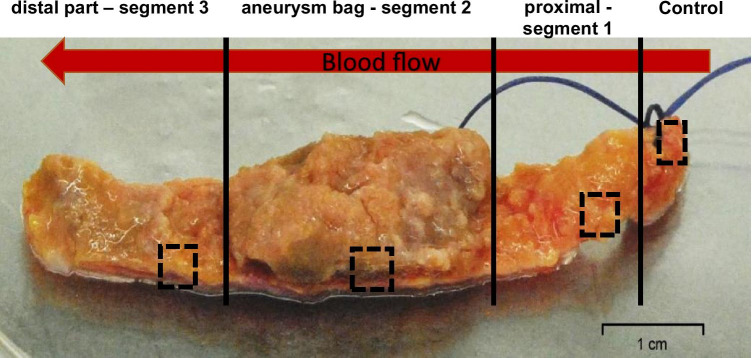
Abdominal aortic aneurysm collected during an open aortic repair (OAR) schematically showing the plan of partitioning of the tissue. From each segment, pieces of ~ 4 mm × 4 mm × 2–4 mm were subjected immediately to RNA isolation and purification (boxed)

Fragments of AAA, usually approximately 50 mm long, were collected from the patients upon surgery. Non-aneurysmal aortic samples of the aneurysm neck (unaffected samples) were simultaneously collected for use as controls (Fig. 1).

All surgical procedures were performed in the planned mode. Briefly, the material collected for the research was part of the aneurysm excised during an OAR. The samples were collected intraoperatively in the General and Vascular Surgery Clinic (Katowice-Ochojec, Poland) and secured immediately in the operating room at room temperature in sterile 50 mL tubes filled with 25 mL of Dulbecco’s modified Eagle’s medium (Gibco, Grand Island, NY, USA) supplemented with glucose (4.5 mg/mL) (high-glucose DMEM), penicillin (10,000 U/ml), streptomycin (10 mg/ml), and amphotericin B (25 µg/ml) (PAA Laboratories, Pasching, Austria). The procedures were established to maintain living cells because the specimens were also used for cell isolation and their culture to characterize the cell types in the separated layers of the AAA wall. Immediately upon arrival to the cell culture facility, the aneurysm was divided into 4 fragments, border, control/border (C); neck, upper/proximal (1); aneurysm bag, middle/central (2); and the end segment, bottom/distal region (3) (Ziaja 2013), where the second part was the aneurysm sack of the excised AAA. From the fragments, specimens of ~ 4 mm × 4 mm × 2–4 mm were immediately subjected to RNA isolation and purification (Fig. 1).

### Methods

Total RNA was isolated in duplicates using Zymogen Quick RNA Mini Prep (Ambion, Austin, Texas, USA) following sample homogenization in TissueLyser II (Qiagen, Venlo, The Netherlands). Quality and quantity evaluation was performed using a NanoDrop 2000 spectrophotometer (Thermo Fisher Scientific, Waltham, Massachusetts, USA). Total RNA (1 to 2 µg) was transcribed using a cDNA transcriptor first-strand cDNA synthesis lit (Roche, Penzberg, Upper Bavaria, Germany) using random hexamers. Expression analyses with Real Time ready Custom Panel 384–96 (configuration no. 100131839; Roche, Penzberg, Upper Bavaria, Germany) and LightCycler 480 Probe Master (Roche) were performed using a LightCycler 480 II (Roche). The genes analysed in this report are listed in Table 1.

**Table 1:** Alphabetical list of genes used in the study with appropriate assay IDs (Roche) and HGNC symbols. Four reference genes are listed at the beginning of the table

No	Assay ID	Human gene symbol	Description
1	141139	*GAPDH*	Glyceraldehyde-3-phosphate dehydrogenase [Source: HGNC Symbol; Acc: 4141]
2	144221	*GUSB*	Glucuronidase, beta [Source: HGNC Symbol; Acc: 4696]
3	102088	*PPIA*	Peptidylprolyl isomerase A (cyclophilin A) [Source: HGNC Symbol; Acc: 9253]
4	102119	*RPL13A*	Small nucleolar RNA, C/D box 32A [Source: HGNC Symbol; Acc: 10159]
5	102984	*ADAMTS1*	ADAM metallopeptidase with thrombospondin type 1 motif 1 [Source: HGNC Symbol; Acc: 217]
6	108591	*ADAMTS8*	ADAM metallopeptidase with thrombospondin type 1 motif, 8 [Source:HGNC Symbol;Acc:224]
7	109363	*ADAMTS13*	ADAM metallopeptidase with thrombospondin type 1 motif, 13 [Source: HGNC Symbol; Acc: 1366]
8	148270	*MMP1*	Matrix metallopeptidase 1 (interstitial collagenase) [Source: HGNC Symbol; Acc: 7155]
9	139230	*MMP2*	Matrix metallopeptidase 2 (gelatinase A, 72 kDa gelatinase, 72 kDa type IV collagenase) [Source: HGNC Symbol; Acc: 7166]
10	103167	*MMP3*	Matrix metallopeptidase 3 (stromelysin 1, progelatinase) [Source: HGNC Symbol; Acc: 7173]
11	104396	*MMP7*	Matrix metallopeptidase 7 (matrilysin, uterine) [Source: HGNC Symbol; Acc: 7174]
12	146302	*MMP8*	Matrix metallopeptidase 8 (neutrophil collagenase) [Source: HGNC Symbol; Acc:7175]
13	136019	*MMP9*	Matrix metallopeptidase 9 (gelatinase B, 92 kDa, gelatinase, 92 kDa, type IV collagenase) [Source: HGNC Symbol; Acc: 7176]
14	108842	*MMP10*	Matrix metallopeptidase 10 (stromelysin 2) [Source: HGNC Symbol; Acc: 7156]
15	148278	*MMP11*	Matrix metallopeptidase 11 (stromelysin 3) [Source: HGNC Symbol; Acc: 7157]
16	tbd	*MMP12*	Matrix metallopeptidase 12 (macrophage elastase) [Source: HGNC Symbol; Acc: 7158]
17	140652	*MMP13*	Matrix metallopeptidase 13 (collagenase 3) [Source: HGNC Symbol; Acc: 7159]
18	109081	*MT1-MMP*	Matrix metallopeptidase 14 (membrane-inserted) [Source: HGNC Symbol; Acc: 7160]
19	108327	*MT2-MMP*	Matrix metallopeptidase 15 (membrane-inserted) [Source: HGNC Symbol; Acc: 7161]
20	108880	*MT3-MMP*	Matrix metallopeptidase 16 (membrane-inserted) [Source: HGNC Symbol; Acc: 7162]
21	147557	*TIMP1*	TIMP metallopeptidase inhibitor 1 [Source: HGNC Symbol; Acc: 11820]
22	110664	*TIMP2*	TIMP metallopeptidase inhibitor 2 [Source: HGNC Symbol; Acc: 11821]
23	101221	*TIMP3*	TIMP metallopeptidase inhibitor 3 [Source: HGNC Symbol; Acc: 11822]
24	112044	*TIMP4*	TIMP metallopeptidase inhibitor 4 [Source: HGNC Symbol; Acc: 11823]

### Gene expression profiling

Gene expression was analysed using GenEx ver6 software (MultiD analyses AB; Göteborg, Szwecja). Raw data were subjected to normalization to sample amount followed by normalization to the reference genes *GAPDH*, *GusB*, *PPIA*, and *RPL13a* (Table 1). Relative expression of target genes (ΔΔCt) was calculated with the comparison against the control/border samples. The last preprocessing step was filling the missing data with 0.

### Statistical analyses

The Kolmogorov–Smirnov test was employed to determine if the data from the expression analysis showed a normal distribution. Only for the *MMP11* the distribution was normal. Due to the small sample group size, the data for the analysis were based on the calculations of the median and sem (Weissgerber et al. 2015). In the case of not normally distributed data, the non-parametric test (one-tailed Mann–Whitney test) was used for data analysis. The threshold for the p-value was set to less than 0.05. For *MMP11* analysis, a one-tailed t-test was used with the p-value set to less than 0.05. For the determination of the differential expression of genes, scatter plot analysis was used with a significance area equal to 1. Spearman correlation coefficients (r_S_) were calculated to determine the correlation between genes (Online Resource 1).

## Results

### Expression of mRNA in AAA tissues

An attempt was made to isolate RNA from all fragments in 29 patients. Unfortunately, the quantity and quality of the isolated total RNA from all samples were neither satisfying nor sufficient for effective analysis by RT-qPCR. The RNA was successfully isolated only from samples of 14 patients and it was not always possible to get biological replicates. For the same reasons, the study group did not include tissues from women but only from men (Online Resource 2).

Using total RNA extracted from 7 border tissues, 13 from the proximal and middle part of AAA, and 20 from the distal part, RT-PCR was performed to detect the presence and relative expression of specific mRNA. The mRNA of the analysed genes was detected in all parts of AAA, except for *MMP10* and *ADAMTS13*. For the two genes, the expression was detected in less than 40% of the samples. Additionally, in the case of *MMP10*, there was no expression of this gene in the proximal part of AAAs (Online Resource 3).

### Differential mRNA expression of extracellular matrix enzymes in AAA segments

**Fig. 2:**
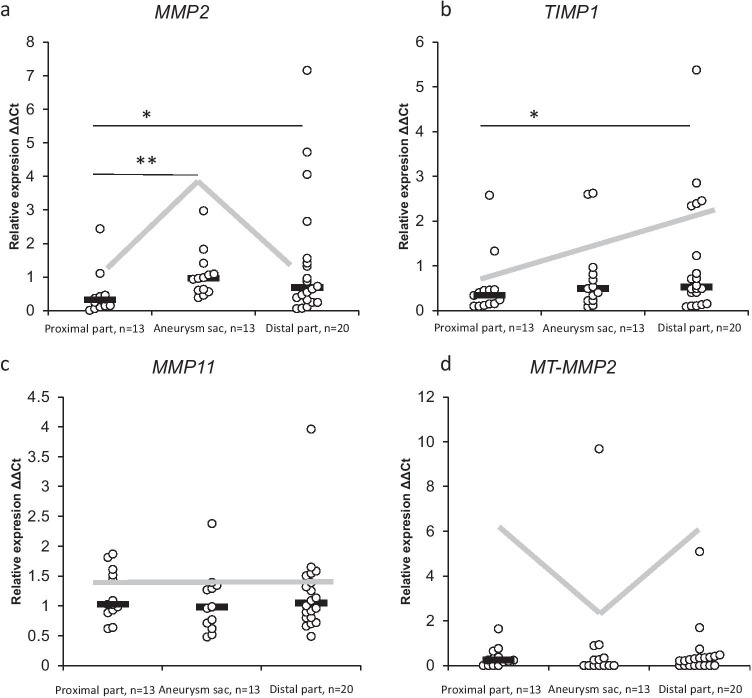
Relative expression of selected genes encoding matrix metalloproteinases and matrix metalloprotease inhibitors in aneurysm and surrounding tissues representing the four gene groups. Non-parametric Mann–Whitney test and T-test (for MMP11) were performed (* *p* < 0.05, ** *p* < 0.05). a, group I; b, group II; c, group III; and d, group IV. The grey line represents the trend in the expression gradient

To investigate the differences between ECM enzymes, the relative expression of genes in the aneurysm sac and surrounding segments was measured. Based on the distribution of expression between pathological tissue and the border, genes could be clustered into four groups (Fig. 2 and Online Resource 4).

Group I (Online Resource 5) is constituted of genes showing higher expression in the aneurysm sac than in the adjacent tissues. Proangiogenic *MMP2*, *MT-MMP1*, and *TIMP2*, known to be associated with ECM remodelling, were more abundant in the aneurysm sac and significantly differed from the expression in proximal segments. In group II (Online Resource 6) were genes that revealed decreased expression in the distal when compared to the proximal part. *MMP2* and *TIMP1* have been identified as strong proangiogenic and anti-apoptotic factors and showed significant differences between the two segments of the analysed tissues. Group III (Online Resource 7) included genes with no changes in the expression between the analysed segments. In group IV were genes which revealed lower expression in pathological tissue than in surrounding tissues. The antiangiogenic factor *MT-MMP2* and proteins expressed by depleted VSMC (e.g. *MMP8* and *MMP13*) showed the lowest expression in the aneurysm sac in comparison to adjacent tissues.

The highest fold change between proximal fragment and aneurysm sac was observed for *TIMP4* (3.82 ± 0.002), and no differences have been detected between those fragments for *TIMP1*, *MMP12*, *MMP10*, *MMP11*, *ADAMTS13*, and *MMP8* (Table 2). Smaller differences in expression of most analysed genes were observed between the aneurysm sac and the distal part. The greatest differences were found here for *ADAMTS8* (2.92 ± 0.13). No differences in expression were detected for *MMP1*, *MMP7*, *MMP9*, *MMP10*, *MMP11*, *MMP12*, *MT-MMP1*, *MT-MMP3*, *ADAMTS13*, *TIMP2*, and *TIMP3*). Significantly large changes in the expression of the analysed genes were observed between the proximal and distal parts, with the greatest difference for MMP3 (-4.33 ± 0.41). No expression changes were detected for *MMP8*, *MMP10*, *MMP11*, *MMP13*, *MT-MMP3*, *MT-MMP2*, *ADAMTS1*, *ADAMTS8*, and *ADAMTS13*.

**Table 2:** Fold change of expression between parts of aneurysm

Gene	Proximal vs aneurysm sac	Aneurysm sac vs distal part	Proximal vs distal
Group I			
* TIMP4*	-3,82169**	2,19975	-1,73733*
* ADAMTS8*	-3,39204	2,92517	-1,1596
* ADAMTS1*	-2,28174	2,20776	-1,03351
* TIMP3*	-2,25861*	-1,06421	-2,40363*
* MMP1*	-1,92413	-1,07369	-2,06593
* MMP7*	-1,89143	1,2481	-1,51544
* MMP3*	-1,72753	-2,50842	-4,33338
* MMP2*	-1,50789*	-1,28329	-1,93506*
* MT-MMP1*	-1,48615*	-1,21837	-1,81067*
* TIMP2*	-1,46273**	1,03951	-1,40714
* MT-MMP3*	-1,29784*	1,10869	-1,1706
Group II			
* MMP9*	-1,65547	-1,06107	-1,75657*
* TIMP1*	-1,18729	-1,26522	-1,50218*
* MMP12*	-1,13957	-1,17196	-1,33552*
Group III			
* MMP10*	-1,05705	1,00192	-1,05502
* MMP11*	1,09707	-1,1213	-1,02208
* ADAMTS13*	1,11656	-1,0644	1,04901
GROUP IV			
* MT-MMP2*	-1,51556	1,34533	-1,12654
* MMP8*	1,10681	-1,27731	-1,15405
* MMP13*	1,91792	-1,85845	1,032

Statistically significant data are marked in bold. Non-parametric Mann–Whitney test and T-test (for *MMP11*) were performed (* *p* < 0.05, ** *p* < 0.05)

## Discussion

Our study carefully examined the expression of 20 genes encoding matrix metalloproteinases and their inhibitors. It is commonly accepted that MMP and TIMP overexpression is the major factor in aneurysm progression. Studies by others on profiling of the expression of 16 genes encoding MMPs revealed their elevated levels in AAAs, but the only significantly higher expressed was *MMP9* (Armstrong et al. 2002). Additionally, no changes were detected in TIMP expression. Reports by others also showed the overexpression of mRNA and higher concentration of protein encoded by *MMP12* in AAA tissues (Curci et al. 1998). At the protein level, also, increased content of *MMP1* and *MMP9* as well as *TIMP2* was found (Koullias et al. 2004).

Abdominal aortic aneurysms are not the same; thus, the differences between the aneurysm’s segments make them difficult to interpret. Researchers were mostly focused on the differences between affected tissues and their normal counterparts from control non-AAA donors. To overcome the difficulties caused by heterogeneity of AAA structures, some assessments were performed using DENSE cardiovascular magnetic resonance or analyses of the regional distribution of aortic wall thickness (Raghavan et al. 2006; Wilson et al. 2019). However, no correlations of gene expression profiles were conducted. Proximal to distal aneurysm analyses usually were performed in the clinic, but no extensive basic research was conducted (Polanczyk et al. 2019). Analysis of the distribution of expression along the AAAs presented in this work gave a better understanding of the processes involved in the progression of aneurysms. The margin, non-aneurysmal aortic samples was assumed as control/reference tissue, which allowed to further elucidate the molecular base of the development and progression of AAA.

Here, the expression of mRNAs encoding metalloproteinases and their inhibitors was found to be irregular and variable along AAA. For example, the ratio *MMP1*:*TIMP1* mRNAs, which differed between segments (from 0.85 in the proximal part, nearly doubled (1.65) in aneurysm sac and 1.1 in the distal segment) could be a result of dysregulation of their expression in the segments. Moreover, only moderate correlation (r_S_ = 0.41) was found between the relative expression of *MMP1* and *TIMP1* along the aneurysm tissue. Soluble protease, *MMP1*, breaks down interstitial collagens, including types I and III, both present in the arterial walls. *MMP1* is only inhibited by *TIMP1*, and therefore, the regulation of the two gene products is critical for proper matrix remodelling.

In the inception of angiogenesis, the genes encoding *MMP2*, *MMP9*, *MT1-MMP*, *TIMP1*, and *TIMP2* play key roles (Safina et al. 2007). The very strong to almost complete correlation of expression between genes involved in the onset of angiogenesis suggests that the regulation of angiogenesis occurred in the AAA border tissue (Online Resource 1). Therefore, as the abnormal tissue deteriorates, the surrounding tissue compensates for the lack of oxygen and nutrients in the affected tissue, by exporting the products to the ECM.

Proteolytic degradation of the ECM in the aneurysm wall is mainly governed by active *MMP2* and *MMP9*, whose genes showed different expression patterns, with the highest peak in the aneurysm sack in *MMP2* and in the distal part in *MMP9*.

Variable expression of genes encoding the 3 collagenases *MMP1*, *MMP3*, and *MMP13* in the AAAs was observed. The lowest expression of *MMP13* was detected in the aneurysm sac. For *MMP1* and *MMP3*, the lowest expression was in the proximal part. *MMP1* is primarily secreted by mesenchymal cells such as vascular smooth muscle cells (VSMCs) or fibroblasts and is activated by *MMP3* and the urokinase plasmin activator (uPA)/plasmin system. Other groups also reported lower expression of *TIMP1* than *MMP1* (Knox et al. 1997). VSMCs express *MMP13*, which is consistent with the fact that VSMCs are depleted in aneurysms, leading to a lack of collagenases needed for proteolysis of the excess of collagen in the medial and adventitial layers (Kadoglou and Liapis 2004).

The involvement of these MMPs in angiogenesis primarily relates to the degradation of ECM, but it should be noted that the activities of these proteases are complex and may involve other effects, such as the activation of growth factors and cytokinesis, the recruitment of endothelial progenitor cells, and the degradation of inhibitors (Kadoglou and Liapis 2004; Safina et al. 2007). Tissue remodelling involving proteolysis is only one of the critical steps in angiogenesis. Excessive proteolysis leads to damage of the blood vessel and can promote ECM decay, preventing cell migration instead of their attachment to it. In healthy blood vessels, there is no or very low expression of matrix metalloproteinases.

The AAA inflammatory process involves macrophages producing pro*MMP12* and activating the proteolytic degradation of ECM depending on the action of *MMP2* and *MMP9*. The process is then dispersed on both sides of the focal point of inflammation. As aneurysm enlarges, apoptosis of endothelial cells and expansion of VSMCs occur, leading to the overexpression of proangiogenic enzymes. In response to the overexpression of *MMP2* and *MMP9* under the influence of *MMP12*, antiangiogenic enzymes start to balance their expression, which leads to their overexpression. Moreover, in AAA, the overgrowth of VSMCs produces the ECM surplus, which leads to nutrient and oxygen deficiency. The tissue that surrounds the aneurysm must compensate for those conditions by ECM proteolysis and angiogenesis toward overgrown tissue, thus overexpressing metalloproteinases that are transported in the ECM toward the focal point of the AAA. The excess of proangiogenic proteases is counteracted by inhibitors that presumably arise from the central part of the developing aneurysm. The highest expression of genes encoding *TIMP4* and other inhibitors in aneurysm sac is the strong evidence for this postulate. *TIMP4* expressed by VSMCs is linked to pathological inflammation engaging ECM remodelling (Koskivirta et al. 2006).

In conclusion, the report delivers new evidence on the differential expression patterns of selected genes involved in ECM synthesis and its remodelling between AAA segments. Results provide insight into the complex interplay between MMPs and their inhibitors as well as between the process that aortic vessels undergo during the formation of AAA. The molecular mechanisms underlying the AAA progression needs further to work out all possible interactions in AAA segments but also in particular layers of aberrant aortic vessel walls.

## Supplementary Information

Below is the link to the electronic supplementary material.

Supplementary file1 (PDF 162 KB)

Supplementary file2 (PDF 137 KB)

Supplementary file3 (PDF 79 KB)

Supplementary file4 (PDF 88 KB)

Supplementary file5 (PDF 195 KB)

Supplementary file6 (PDF 138 KB)

Supplementary file7 (PDF 139 KB)

Supplementary file8 (PDF 138 KB)

## Data Availability

The data that support the findings of this study are available from the corresponding author upon reasonable request.
