# Supplementary material for: Expression gradient of metalloproteinases and their inhibitors from proximal to distal segments of abdominal aortic aneurysm
Source: J Appl Genet. 2021 Jun 6;62(3):499–506. doi: 10.1007/s13353-021-00642-3 (PMC8357691; doi:10.1007/s13353-021-00642-3)
Supplement: Supplementary file 1 — Supplementary file1 (PDF 162 KB) [file 13353_2021_642_MOESM1_ESM.pdf]

“Expression gradient of metalloproteinases and their inhibitors from proximal to distal segments of abdominal aortic aneurysm”
Journal of Applied Genetics
Aleksandra Auguściak-Duma, Karolina L. Stępień, Marta Lesiak, Ewa Gutmajster, Agnieszka Fus-Kujawa, Malwina Botor, Aleksander L. Sieroń
Corresponding author: Aleksandra Auguściak-Duma, Department of Molecular Biology, Faculty of Medical Science in Katowice, Medical University of Silesia, Katowice, Poland. E-mail: aausgusciak@sum.edu.pl (AAD). ORCID-0000-0001-5426-3277

Online Resource 1 Spearman coefficient

	ADAMTS1	ADAMTS8	ADAMTS13	MMP1	MMP2	MMP3	MMP7	MMP8	MMP9	MMP10	MMP11	MMP12	MMP13	MMP14	MMP15	MMP16	TIMP1	TIMP2	TIMP3	TIMP4
ADAMTS1	1,00	0,47	0,52	0,07	0,63	0,42	0,09	-0,66	0,29	0,35	-0,58	0,21	0,41	0,41	0,35	0,68	0,41	0,79	0,66	0,50
ADAMTS8	0,47	1,00	0,45	0,37	0,59	0,23	0,33	-0,51	0,60	0,09	-0,48	0,38	0,25	0,47	0,41	0,58	0,50	0,65	0,46	0,36
ADAMTS13	0,52	0,45	1,00	0,34	0,61	0,33	0,34	-0,56	0,34	0,10	-0,51	0,19	0,47	0,56	0,60	0,49	0,48	0,60	0,55	0,47
MMP1	0,07	0,37	0,34	1,00	0,48	0,20	0,56	-0,36	0,45	0,30	-0,17	0,49	0,24	0,45	0,31	0,18	0,41	0,40	0,32	0,33
MMP2	0,63	0,59	0,61	0,48	1,00	0,29	0,44	-0,54	0,64	0,31	-0,56	0,44	0,47	0,86	0,59	0,67	0,73	0,93	0,91	0,66
MMP3	0,42	0,23	0,33	0,20	0,29	1,00	0,12	-0,26	0,11	0,06	-0,28	0,18	0,36	0,23	0,06	0,38	0,21	0,37	0,30	0,10
MMP7	0,09	0,33	0,34	0,56	0,44	0,12	1,00	-0,25	0,56	0,27	0,01	0,51	0,28	0,56	0,32	0,22	0,45	0,38	0,38	0,36
MMP8	-0,66	-0,51	-0,56	-0,36	-0,54	-0,26	-0,25	1,00	-0,43	-0,31	0,72	-0,33	-0,45	-0,45	-0,29	-0,51	-0,58	-0,62	-0,53	-0,55
MMP9	0,29	0,60	0,34	0,45	0,64	0,11	0,56	-0,43	1,00	0,30	-0,45	0,72	0,39	0,63	0,38	0,39	0,67	0,61	0,61	0,41
MMP10	0,35	0,09	0,10	0,30	0,31	0,06	0,27	-0,31	0,30	1,00	-0,09	0,26	0,29	0,34	0,20	0,18	0,24	0,32	0,31	0,43
MMP11	-0,58	-0,48	-0,51	-0,17	-0,56	-0,28	0,01	0,72	-0,45	-0,09	1,00	-0,31	-0,36	-0,46	-0,33	-0,43	-0,52	-0,64	-0,57	-0,39
MMP12	0,21	0,38	0,19	0,49	0,44	0,18	0,51	-0,33	0,72	0,26	-0,31	1,00	0,43	0,45	0,08	0,30	0,63	0,45	0,39	0,30
MMP13	0,41	0,25	0,47	0,24	0,47	0,36	0,28	-0,45	0,39	0,29	-0,36	0,43	1,00	0,52	0,18	0,49	0,40	0,48	0,51	0,45

MMP14	0,41	0,47	0,56	0,45	0,86	0,23	0,56	-0,45	0,63	0,34	-0,46	0,45	0,52	1,00	0,60	0,55	0,73	0,76	0,81	0,69
MMP15	0,35	0,41	0,60	0,31	0,59	0,06	0,32	-0,29	0,38	0,20	-0,33	0,08	0,18	0,60	1,00	0,37	0,38	0,56	0,53	0,40
MMP16	0,68	0,58	0,49	0,18	0,67	0,38	0,22	-0,51	0,39	0,18	-0,43	0,30	0,49	0,55	0,37	1,00	0,45	0,75	0,64	0,46
TIMP1	0,41	0,50	0,48	0,41	0,73	0,21	0,45	-0,58	0,67	0,24	-0,52	0,63	0,40	0,73	0,38	0,45	1,00	0,65	0,71	0,53
TIMP2	0,79	0,65	0,60	0,40	0,93	0,37	0,38	-0,62	0,61	0,32	-0,64	0,45	0,48	0,76	0,56	0,75	0,65	1,00	0,89	0,62
TIMP3	0,66	0,46	0,55	0,32	0,91	0,30	0,38	-0,53	0,61	0,31	-0,57	0,39	0,51	0,81	0,53	0,64	0,71	0,89	1,00	0,65
TIMP4	0,50	0,36	0,47	0,33	0,66	0,10	0,36	-0,55	0,41	0,43	-0,39	0,30	0,45	0,69	0,40	0,46	0,53	0,62	0,65	1,00

Online resource 1 Spearman coefficient p-value

	ADAMTS1	ADAMTS8	ADAMTS13	MMP1	MMP2	MMP3	MMP7	MMP8	MMP9	MMP10	MMP11	MMP12	MMP13	MMP14	MMP15	MMP16	TIMP1	TIMP2	TIMP3	TIMP4
ADAMTS1	0,00	0,00	0,00	0,62	0,00	0,00	0,53	0,00	0,04	0,01	0,00	0,14	0,00	0,00	0,01	0,00	0,00	0,00	0,00	0,00
ADAMTS8	0,00	0,00	0,00	0,01	0,00	0,09	0,02	0,00	0,00	0,53	0,00	0,01	0,07	0,00	0,00	0,00	0,00	0,00	0,00	0,01
ADAMTS13	0,00	0,00	0,00	0,01	0,00	0,02	0,01	0,00	0,01	0,49	0,00	0,18	0,00	0,00	0,00	0,00	0,00	0,00	0,00	0,00
MMP1	0,62	0,01	0,01	0,00	0,00	0,15	0,00	0,01	0,00	0,03	0,22	0,00	0,08	0,00	0,03	0,20	0,00	0,00	0,02	0,02
MMP2	0,00	0,00	0,00	0,00	0,00	0,04	0,00	0,00	0,00	0,03	0,00	0,00	0,00	0,00	0,00	0,00	0,00	0,00	0,00	0,00
MMP3	0,00	0,09	0,02	0,15	0,04	0,00	0,40	0,06	0,42	0,67	0,04	0,19	0,01	0,10	0,67	0,01	0,14	0,01	0,03	0,49
MMP7	0,53	0,02	0,01	0,00	0,00	0,40	0,00	0,07	0,00	0,05	0,95	0,00	0,04	0,00	0,02	0,12	0,00	0,00	0,01	0,01
MMP8	0,00	0,00	0,00	0,01	0,00	0,06	0,07	0,00	0,00	0,02	0,00	0,02	0,00	0,00	0,04	0,00	0,00	0,00	0,00	0,00
MMP9	0,04	0,00	0,01	0,00	0,00	0,42	0,00	0,00	0,00	0,03	0,00	0,00	0,00	0,00	0,01	0,00	0,00	0,00	0,00	0,00

MMP10	0,01	0,53	0,49	0,03	0,03	0,67	0,05	0,02	0,03	0,00	0,51	0,06	0,03	0,01	0,16	0,19	0,08	0,02	0,02	0,00
MMP11	0,00	0,00	0,00	0,22	0,00	0,04	0,95	0,00	0,00	0,51	0,00	0,02	0,01	0,00	0,01	0,00	0,00	0,00	0,00	0,00
MMP12	0,14	0,01	0,18	0,00	0,00	0,19	0,00	0,02	0,00	0,06	0,02	0,00	0,00	0,00	0,56	0,03	0,00	0,00	0,00	0,03
MMP13	0,00	0,07	0,00	0,08	0,00	0,01	0,04	0,00	0,00	0,03	0,01	0,00	0,00	0,00	0,19	0,00	0,00	0,00	0,00	0,00
MMP14	0,00	0,00	0,00	0,00	0,00	0,10	0,00	0,00	0,00	0,01	0,00	0,00	0,00	0,00	0,00	0,00	0,00	0,00	0,00	0,00
MMP15	0,01	0,00	0,00	0,03	0,00	0,67	0,02	0,04	0,01	0,16	0,01	0,56	0,19	0,00	0,00	0,01	0,00	0,00	0,00	0,00
MMP16	0,00	0,00	0,00	0,20	0,00	0,01	0,12	0,00	0,00	0,19	0,00	0,03	0,00	0,00	0,01	0,00	0,00	0,00	0,00	0,00
TIMP1	0,00	0,00	0,00	0,00	0,00	0,14	0,00	0,00	0,00	0,08	0,00	0,00	0,00	0,00	0,00	0,00	0,00	0,00	0,00	0,00
TIMP2	0,00	0,00	0,00	0,00	0,00	0,01	0,00	0,00	0,00	0,02	0,00	0,00	0,00	0,00	0,00	0,00	0,00	0,00	0,00	0,00
TIMP3	0,00	0,00	0,00	0,02	0,00	0,03	0,01	0,00	0,00	0,02	0,00	0,00	0,00	0,00	0,00	0,00	0,00	0,00	0,00	0,00
TIMP4	0,00	0,01	0,00	0,02	0,00	0,49	0,01	0,00	0,00	0,00	0,00	0,03	0,00	0,00	0,00	0,00	0,00	0,00	0,00	0,00
