# Supplementary material for: Expression gradient of metalloproteinases and their inhibitors from proximal to distal segments of abdominal aortic aneurysm
Source: J Appl Genet. 2021 Jun 6;62(3):499–506. doi: 10.1007/s13353-021-00642-3 (PMC8357691; doi:10.1007/s13353-021-00642-3)
Supplement: Supplementary file 2 — Supplementary file2 (PDF 137 KB) [file 13353_2021_642_MOESM2_ESM.pdf]

“Expression gradient of metalloproteinases and their inhibitors from proximal to distal segments of abdominal aortic aneurysm”

Journal of Applied Genetics

Aleksandra Auguściak-Duma, Karolina L. Stępień, Marta Lesiak, Ewa Gutmajster, Agnieszka Fus-Kujawa, Malwina Botor, Aleksander L. Sieroń

Corresponding author: Aleksandra Auguściak-Duma, Department of Molecular Biology, Faculty of Medical Science in Katowice, Medical University of Silesia, Katowice, Poland. E-mail: aaugusciak@sum.edu.pl (AAD). ORCID-0000-0001-5426-3277

Online Resource 2 Summary of age and sex of patients who were donors of AAAs samples for cells isolation and characterization and from whose samples the mRNA was purified at quantities and quality satisfying for gene expression profiling

No.	Patient No.	Sex	Age [years]	Availability of biological replicates
1.	**T14**	M	66	Yes, for aneurysm sac and distal part
2.	**T15**	M	65	N.A.
3.	**T16**	M	72	N.A.
4.	**T19**	M	70	Yes, for all parts of extracted aneurysm
5.	**T20**	M	58	N.A.
6.	**T21**	M	57	Yes, for proximal part
7.	**T22**	M	73	N.A.
8.	**T23**	M	82	Yes, for distal part and control tissue
9.	**T24**	M	65	Yes, for proximal part and distal part
10.	**T25**	M	65	Yes, for distal part and control tissue
11.	**T26**	M	65	Yes, for all parts of the extracted aneurysm and control tissue
12.	**T27**	M	71	N.A.
13.	**T28**	M	65	N.A.
14.	**T29**	M	71	Yes, for aneurysm sac
	**Mean ± S.D.**		**67.5 ± 6.35**	

M – male; S.D. – standard deviation; N.A. – material was not available for biological replicate
