# Supplementary material for: Expression gradient of metalloproteinases and their inhibitors from proximal to distal segments of abdominal aortic aneurysm
Source: J Appl Genet. 2021 Jun 6;62(3):499–506. doi: 10.1007/s13353-021-00642-3 (PMC8357691; doi:10.1007/s13353-021-00642-3)
Supplement: Supplementary file 3 — Supplementary file3 (PDF 79 KB) [file 13353_2021_642_MOESM3_ESM.pdf]

“Expression gradient of metalloproteinases and their inhibitors from proximal to distal segments of abdominal aortic aneurysm”

Journal of Applied Genetics

Aleksandra Auguściak-Duma, Karolina L. Stępień, Marta Lesiak, Ewa Gutmajster, Agnieszka Fus-Kujawa, Malwina Botor, Aleksander L. Sieroń

Corresponding author: Aleksandra Auguściak-Duma, Department of Molecular Biology, Faculty of Medical Science in Katowice, Medical University of Silesia, Katowice, Poland. E-mail: aaugusciak@sum.edu.pl (AAD). ORCID-0000-0001-5426-3277

Online Resource 3 Gene expression detected at the percentage of the analysed AAA segments

Gene	Control	Proximal part	Aneurysm bag	Distal part
n=	7	13	13	20
ADAMTS1	100	100	100	100
ADAMTS8	100	77	77	60
ADAMTS13	57	38	31	30
MMP1	100	77	85	90
MMP2	100	100	100	100
MMP3	71	62	77	65
MMP7	100	77	77	80
MMP8	100	100	100	100
MMP9	100	100	92	95
MMP10	29	0	15	20
MMP11	100	100	100	100
MMP12	100	100	77	100
MMP13	86	62	77	75
MT1-MMP	100	100	100	100
MT2-MMP	71	69	46	60
MT3-MMP	100	92	100	85
TIMP1	100	100	100	100
TIMP2	100	100	100	100
TIMP3	100	100	100	100
TIMP4	86	77	92	80
