# Supplementary material for: Expression gradient of metalloproteinases and their inhibitors from proximal to distal segments of abdominal aortic aneurysm
Source: J Appl Genet. 2021 Jun 6;62(3):499–506. doi: 10.1007/s13353-021-00642-3 (PMC8357691; doi:10.1007/s13353-021-00642-3)
Supplement: Supplementary file 4 — Supplementary file4 (PDF 88 KB) [file 13353_2021_642_MOESM4_ESM.pdf]

“Expression gradient of metalloproteinases and their inhibitors from proximal to distal segments of abdominal aortic aneurysm”

Journal of Applied Genetics

Aleksandra Auguściak-Duma, Karolina L. Stępień, Marta Lesiak, Ewa Gutmajster, Agnieszka Fus-Kujawa, Malwina Botor, Aleksander L. Sieroń
Corresponding author: Aleksandra Auguściak-Duma, Department of Molecular Biology, Faculty of Medical Science in Katowice, Medical University of Silesia, Katowice, Poland. E-mail: aaugusciak@sum.edu.pl (AAD). ORCID-0000-0001-5426-3277

Online Resource 4 Median of relative expression of analysed genes with statistical analysis. Non-parametric Mann-Whitney test and T-test (for *MMP11*) were performed (* p <0.05, ** p <0.05)

Median of relative expression				Mann-Whitney p-value		
**Gene**	**Proximal part**	**Aneurysm sac**	**Distal part**	**Proximal vs Aneurysm sac**	**Aneurysm sac vs Distal Part**	**Proximal vs Distal**
**GROUP I**						
MMP7	0,09665	0,11316	0,09741	0,17798	0,25954	0,39115
ADAMTS8	0,18089	0,27609	0,11451	0,27768	0,13056	0,30946
MT-MMP3	0,08224	0,32841	0,18079	0,02275*	0,13056	0,11920
TIMP4	0,06895	0,42166	0,35938	0,00221**	0,22504	0,04333*
TIMP2	0,15651	0,51291	0,26926	0,00240**	0,13056	0,06783
ADAMTS1	0,30377	0,51982	0,20598	0,06197	0,10855	0,39115
MMP3	0,52814	0,69568	0,16778	0,38895	0,30299	0,41252
MMP1	0,29423	0,79278	0,63120	0,08309	0,44870	0,05869
TIMP3	0,31449	0,86818	0,69802	0,00599*	0,19329	0,03140*
MT-MMP1	0,31864	0,89813	0,77017	0,00599*	0,49265	0,00791*
MMP2	0,32963	0,96522	0,69028	0,00124**	0,23060	0,02044*
**GROUP II**						
MMP12	0,05569	0,11313	0,18499	0,25249	0,34264	0,04683*
MMP9	0,12741	0,24914	0,25234	0,09991	0,39824	0,02888*
TIMP1	0,34176	0,49778	0,53048	0,12962	0,33589	0,04005*
**GROUP III**						
MMP10	0,00000	0,00000	0,00000	0,26075	0,43416	0,17373
ADAMTS13	0,00000	0,00000	0,00000	0,28634	0,39115	0,40536
MMP11[1]	1,02532	0,98015	1,04501	0,23513[1]	0,24135[1]	0,44440[1]
**GROUP IV**						
MT-MMP2	0,23269	0,00000	0,23351	0,34081	0,32256	0,49265
MMP13	0,20365	0,15434	0,44428	0,48977	0,06545	0,16905
MMP8	1,56297	1,21359	1,36659	0,08309	0,14685	0,34943

[1] – T-test, 1-tailed, p-value
