# Supplementary material for: Expression gradient of metalloproteinases and their inhibitors from proximal to distal segments of abdominal aortic aneurysm
Source: J Appl Genet. 2021 Jun 6;62(3):499–506. doi: 10.1007/s13353-021-00642-3 (PMC8357691; doi:10.1007/s13353-021-00642-3)

“Expression gradient of metalloproteinases and their inhibitors from proximal to distal segments of abdominal aortic aneurysm”

Journal of Applied Genetics

Aleksandra Auguściak-Duma, Karolina L. Stępień, Marta Lesiak, Ewa Gutmajster, Agnieszka Fus-Kujawa, Malwina Botor, Aleksander L. Sieroń

Corresponding author: Aleksandra Auguściak-Duma, Department of Molecular Biology, Faculty of Medical Science in Katowice, Medical University of Silesia, Katowice, Poland. E-mail: aausgusciak@sum.edu.pl (AAD). ORCID-0000-0001-5426-3277

Online Resource 6 Relative expression of analysed genes encoding matrix metalloproteinases and matrix metalloprotease inhibitors in aneurysm and surrounding tissues assigned to Group II. Non-parametric Mann-Whitney test was performed (* p <0.05, ** p <0.05).

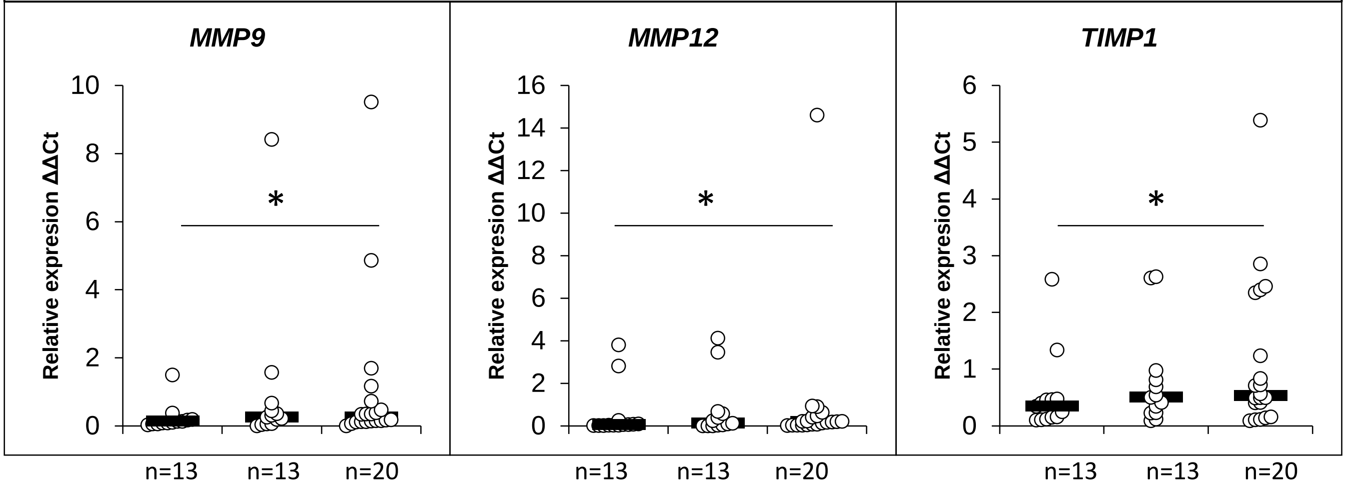

Supplement: Supplementary file 6 — Supplementary file6 (PDF 138 KB) [file 13353_2021_642_MOESM6_ESM.pdf]
